# A Narrative Review of Emerging Therapies for Hypertrophic Obstructive Cardiomyopathy

**DOI:** 10.2174/1573403X19666230324102828

**Published:** 2023-07-05

**Authors:** Antonio da Silva Menezes Junior, Ana Ligia Valeriano de Oliveira, Thais Aratak Maia, Silvia Marçal Botelho

**Affiliations:** 1 Internal Medicine Department, Medicine School, Federal University of Goiás, Goiânia, Goiás, Brazil;; 2 Medical School, Pontifical Catholic University of Goiás, Goiânia, Goiás, Brazil

**Keywords:** Hypertrophic obstructive cardiomyopathy, therapeutics, alcohol septal ablation, surgical myectomy, radiofrequency septal ablation, comorbidities

## Abstract

Hypertrophic obstructive cardiomyopathy is a hereditary condition that affects myocardial contraction. In case of failure of pharmacological treatment, alternative approaches might be used that include surgical myectomy, percutaneous transluminal septal myocardial ablation, and radiofrequency ablation. In respect of long-term advantages, surgical septal myectomy remains the therapy of choice for symptomatic hypertrophic obstructive cardiomyopathy. Alcohol septal ablation has been considered an alternative to surgical myectomy, which confers the benefits of a shorter hospital stay, less discomfort, and fewer complications. However, only expert operators should perform it on carefully chosen patients. Further, radiofrequency septal ablation reduces the left ventricular outflow tract gradient and improves the NYHA functional class of patients with hypertrophic obstructive cardiomyopathy, despite complications like cardiac tamponade and atrioventricular block. Further research with a larger sample size is required to compare the radiofrequency approach with established invasive treatment methods for hypertrophic obstructive cardiomyopathy. Septal myectomy has low morbidity and mortality rates, making it the preferred procedure; however, the efficacy and morbidity remain debatable. Advances in invasive techniques, including percutaneous septal radiofrequency ablation and transcatheter myotomy, have provided alternative approaches for reducing left ventricular outflow tract (LVOT) obstruction in patients who are not candidates for traditional surgical septal myectomy. Candidates for alcohol and radiofrequency septal ablation include patients with symptomatic hypertrophic obstructive cardiomyopathy, older adults, and those with multiple comorbidities.

## 1 INTRODUCTION

Hypertrophic obstructive cardiomyopathy (HOCM) is a cardiac disorder characterized by aberrant fibers that thicken the left ventricular wall, which creates a deficiency in tissue contraction. Heart failure, atrial fibrillation (AF), and even sudden death are potential long-term outcomes of this condition [1, 2]. Hypertrophic cardiomyopathy (HCM) is a common inherited heart disease that affects people of different ethnicities around the world [1, 2].

HCM has several genetic and phenotypic manifestations, and it can have a wide range of clinical repercussions. It is becoming increasingly common to receive a diagnosis of HCM at any age, ranging from infancy to older age. HCM affects one in every 500 people, according to estimates [1]. This incidence increases to one in every 200 with the inclusion of familial transmission, subclinical disease, and pathogenic sarcomere mutations. HCM affects 750,000 individuals in the United States alone. However, only around 100,000 people are clinically diagnosed with HCM each year, implying that the ailment remains underdiagnosed and cardiologists may only see a small number of patients during the course of the disease (the “tip of the iceberg” issue) [3]. HCM has been viewed as a crippling, incurable disease with few effective therapy alternatives since it was first described. Over the last 20 years, improved therapeutic approaches in clinical care for severe complications related to HCM have decreased mortality and morbidity rates and enhanced the average lifespan with excellent quality of life for adults and children [1-4]. Patients with symptoms or those who do not respond to treatment may be identified based on their quality of life. Medications are administered from a practical standpoint [4]. The learning curve for septal myectomy is not a surgical procedure but rather a result of experience and knowledge accumulation, allowing for an excellent left-side ventricular outflow tract reduction without complications, such as ventricular septal defect, aortic valve injury, total atrioventricular block, and the ability to perform a mitral valve repair when necessary [5, 6]. Up to two-thirds of individuals with HCM have left ventricular outflow tract (LVOT) obstruction at rest or due to provocation, which causes debilitating symptoms and an increased risk of sudden death [3-6]. LVOT obstruction occurs due to fast left ventricular ejection *via* a constricted outflow path. The primary treatment for individuals with symptoms is negative inotropic medications that reduce the contractile force. However, many patients respond poorly to maximum therapy and need more extensive septal reduction techniques [7]. Alcohol septal ablation (ASA) is an integrated myectomy that is used as an alternative to surgical myectomy for cardiomyopathy. It was first studied in the middle of the previous century. Using a selective interventricular operation for an obstruction originating from the basal septum (*i.e*., LVOT), it is possible to alleviate the symptoms of a high-objective infarction while simultaneously inducing an infarction with a degree of reduction in LVOT obstruction [7]. Further, ASA is used for patients older than 65 years, those with concomitant diseases, or those with no other treatment options [7, 8]. Therefore, radiofrequency myocardial septal ablation was introduced, an invasive therapeutic choice for drug-resistant HOCM with left ventricular obstruction of less than 50 mmHg, either at rest or after provocation. Considering that it is a less invasive treatment than myectomy and alcohol ablation [9, 10], radiofrequency myocardial septal ablation is often indicated for patients considered to be at high-risk for surgical procedures [10]. This treatment can generate well-defined lesions in the heart using radiofrequency, which induces resistive and conductive heating in the tissues. As the healing progresses, the tissue treated with radiofrequency eventually undergoes involution. The treatment is often conducted under anesthesia, continuously monitoring the patient’s cardiac electrical activity, blood pressure, and oxygen levels. An alternative to radiofrequency myocardial septal ablation is the implantation of a temporary pacemaker. The radiofrequency energy is then delivered through a percutaneous intramyocardial channel guided by computed tomography angiography and echocardiography [9, 10]. The choice of strategy and approach depends on the patient’s symptomatic and clinical differences, as seen in Fig (**1**). Therefore, this choice is crucial as it affects mortality rates, complications, and the overall quality of life. The present study seeks to assess and explain innovative treatments and compare them to more traditional approaches for choosing the best therapy for each case.

## 2 SURGICAL SEPTAL MYECTOMY

Surgical septal myectomy is an established procedure for reducing LVOT obstruction, developed after years of research and progress. Further, it has been proven safe and effective for patients who experience symptoms but do not respond to medical treatment. There are instances when mitral regurgitation or changes in the subvalvular apparatus may be corrected without replacing the mitral valve. However, despite the relative ease of performing surgical septal myectomy, it takes time to learn and accumulate the experience to achieve a good reduction in LVOT obstruction without causing complications, such as ventricular septal defect, aortic valve injury, obstruction atrioventricular valve, and to perform mitral valve repair when necessary [10-14], as illustrated in Fig. (**2**).

The New York Heart Association (NYHA) recommends septal myectomy for patients with heart failure symptoms who are in the functional class III or IV. It has also been shown that septal myectomy may increase the life expectancy of patients with heart failure [11]. A standard procedure for HOCM is transaortic septal myectomy; however, in specific individuals, the left ventricular intracavitary gradient may persist post-surgery as a procedural complication due to the difficulty of the operation [11, 13]. Between October 2011 and July 2014, 676 septal myomectomies were performed at the Mayo Clinic. Among those, 298 consecutive patients were included in the analysis for transaortic procedures. Based on the aforementioned study, AF is the most prevalent consequence after myectomy. In respect of the national statistics, the current study implies that septal myectomy should only be done in centers of excellence with extensive expertise. Only a small proportion of patients previously had myectomy, and AF was the most prevalent complication after surgery [14, 15]. Individuals with symptomatic HOCM with a resting gradient of 50 mmHg or higher are regarded as candidates for ventricular septal myectomy, which is the gold standard for surgical therapy. Those with moderate to severe pulmonary hypertension before surgery and those who underwent myectomy experienced a significant reduction in their peak peripheral arterial pressure [16-18]. Following myectomy, outcomes at three different HCM centers of excellence showed that perioperative mortality was less than 1%, approximately 2-5 percent of patients required definitive pacemaker therapy within 30 days, and reintervention was required in 0-2% of cases during follow-up. In line with the research conducted by Kim *et al.,* it is also widely acknowledged that performing myectomy procedures at high-volume hospitals is associated with decreased rates of mortality and morbidity [19]. Patients diagnosed with HOCM who had undergone myectomy had a decreased all-cause mortality rate and displayed overall survival, which was statistically indistinguishable from those diagnosed with nonobstructive HCM [20]. Using multivariate analysis, myectomy was proven to be a significant and independent determinant of survival. Hence, the variations in long-term survival documented in the current study might be related to surgical improvement in the LVOT gradient [18]. Midventricular obstruction (MVO) is a type of hypertrophic cardiomyopathy (HCM) that is characterized by abnormal thickening of the myocardium in the middle of the left ventricle. It is a rare form of HCM and can lead to severe symptoms such as shortness of breath, chest pain, and fainting. Traditional septal myectomy, which involves removing a portion of the thickened septum, may not be effective in treating MVO. In recent years, transapical septal myectomy (TSM) has emerged as a potential treatment option for MVO. This procedure involves accessing the left ventricle through the apex of the heart and removing the obstructing tissue from the midventricular region. The efficacy of TSM for MVO has been studied in several small case series and retrospective studies. In a study published in the Journal of Thoracic and Cardiovascular Surgery, TSM was performed in 11 patients with MVO who were symptomatic despite medical therapy. The procedure successfully reduced the gradient across the midventricular region in all patients, and there were no significant complications. Follow-up echocardiography showed a sustained improvement in left ventricular function and symptoms over a median follow-up of 25 months [19]. Another study published in the European Journal of Cardio-Thoracic Surgery evaluated the long-term outcomes of TSM in 22 patients with MVO. The procedure successfully reduced the gradient in all patients, and there were no significant complications. At a median follow-up of 60 months, 95% of patients reported an improvement in symptoms, and there was a significant improvement in left ventricular ejection fraction [20]. A recent study published in the Journal of the American College of Cardiology compared the outcomes of TSM with traditional septal myectomy in 92 patients with HCM, including 17 patients with MVO. The study found that TSM was associated with similar procedural success rates and long-term outcomes compared to traditional septal myectomy. TSM may be a suitable alternative for patients with MVO who are not candidates for traditional septal myectomy [21].

## 3 ALCOHOL SEPTAL ABLATION

Although Morrow and colleagues pioneered surgical septal myectomy for HOCM in the 1960s, the condition continues to plague patients [10]. When myectomy is performed in a restricted number of well-established facilities, the results are promising. However, if myectomy is performed in a facility where it is not performed often, it results in a high rate of complications and mortality [6-10]. When Sigwart published his findings in 1995 on a few European patients, he was the first to describe ASA as a minimally invasive procedure for septal reduction that could be conducted through the skin [10-13]. The options for patients with drug-refractory symptoms, who had an obstructive LVOT, included surgical myectomy, ASA, and short atrioventricular delay pacing. The best treatment for LVOT obstruction is still debatable since there are not enough randomized trials that directly examine different medications for the ailment [17-21]. According to recent data, ASA and septal myectomy are not associated with changes in short- and long-term all-cause mortality, cardiovascular mortality, or sudden cardiac death. The method has improved over time, and the outcomes are equivalent to those obtained with myectomy [22].

### 3.1 Development of Ventricular Tachyarrhythmias and Heart Block

LVOT obstruction is associated with the development of heart failure and is a risk factor for sudden cardiac death (SCD) in specific conditions. It has been reported that septal reduction treatments effectively reduce the symptoms and improve survival, which is in line with the general expectations of the population. Since the limitation of LVOT is a substantial factor in the development of ventricular tachyarrhythmias, SCD is predicted to reduce with septal reduction. Further, SCD classification methods have not been tested in patients who underwent septal reduction surgery; however, effective treatment has been found to lower the risk profile for SCD drastically. After septal reduction, patients with previously implanted implantable cardioverter defibrillators (ICDs) had an acceptable shock rate from their ICDs, which is another advantage of successful treatment [23-25]. According to the studies conducted by Rigopoulos *et al.*, percutaneous transluminal septal myocardial ablation (PTSMA) resulted in life-threatening arrhythmic events in only a small percentage of patients with HOCM [26, 27]. Moreover, the HCM Risk-SCD model might predict this outcome [24, 26-30]. The arguments favor this technique because septal ablation is less invasive, the recovery period is shorter, and surgery is more readily available. However, the widespread use of alcohol ablation is concerning since it results in a significant transmural myocardial infarction (on average, 10% of the left ventricular mass and 30% of the septum) and may lead to increased arrhythmogenicity in the heart muscle. Further, ablation is less desirable as a treatment option for myectomy due to the greater risk of complications such as heart block, which necessitates permanent pacing, a less effective decrease in gradient and symptoms, and a shorter follow-up period following the surgery. Notably, 31 studies corroborated these results, and one indicated that just 7 percent of the patients had persistent ventricular tachycardia (VT) or ventricular fibrillation (VF) throughout 30 days. No prolonged VT or VF for more than 30 days was noted after ASA [30-32]. Aksu *et al.* concluded that more patients who underwent ASA needed permanent pacemaker implantation therapy for the entire heart block [32]. According to the findings of Kambiré *et al.*, one-third of the patients experienced non-sustained ventricular tachycardia, 27 patients had an implanted cardioverter defibrillator, and one-third received a new pacemaker for conduction disruption [33]. Consequently, they discovered that ASA was associated with the right bundle branch block in 37–70% of the individuals with the condition. Patients over 55 who underwent alcohol septal ablation were more likely to require a permanent pacemaker following the procedure [34-37]. A study including 243 individuals showed that baseline bundle branch block or VT incidence after 72 hours post-ASA was very low (less than 1.0 percent) [38]. The most prevalent causes of hospital readmissions were AF (12.6 percent), acute systolic heart failure (12.6 percent), paroxysmal ventricular tachycardia (6.4 percent), atrioventricular block (4.9 percent), and heart failure complex (3.0 percent). Individuals with unplanned readmissions following ASA were more likely to encounter a variety of additional health conditions, which majorly comprised of ailments related to the heart [39].

### 3.2 Additional Complication(s)

Koljaja-Batzner *et al.,* previously published a case report on the treatment of a female patient in her sixties with ASA, who was hospitalized for 9 months and had no problems [40-44]. Asil *et al.,* verified no mortality throughout the 12-month follow-up period [44]. Bytyçi *et al.* confirmed their findings, indicating that periprocedural issues were less common with alcohol ablation, even though reintervention and pacemaker implants were more common therein [42-44]. Together, these findings suggest that the transmural myocardial infarction caused by alcohol had a high degree of arrhythmogenicity, which directly resulted from the infarct. Furthermore, the substantial persistence of ventricular arrhythmias after alcohol ablation was observed by Vriesendorp *et al.,* and Cuocco *et al.* They described a large population of patients who underwent alcohol ablation and were implanted with cardioverter-defibrillators [29, 34]. After septal myectomy, the Mayo Clinic observed a notably low incidence of sudden mortality and possibly lethal ventricular tachyarrhythmias as compared to that in other centers [34, 45]. The risk of arrhythmogenicity associated with ASA is a persistent concern [46-48]. Several doctors and HCM specialists, as well as guidelines and consensus committees, raise this issue regularly [49]. Further, the signs and symptoms of ASA also need to be addressed. According to Sossalla *et al.*, a female patient with HOCM aged 78 years was treated with ASA after undergoing acute cardiogenic shock and being unresponsive to extracorporeal membrane oxygenation [50]. However, the patient finally recovered. Olsen and colleagues also hypothesized that decompensated individuals would benefit from urgent alcohol ablation procedures [51]. Considering the limits of myectomy, alcohol ablation may be used as a therapeutic option in patients, especially in older adults or those with comorbidities [52]. According to a literature study by Naidu *et al.*, the outcomes of ASA were comparable with those of surgical myectomy for up to 8–10 years in 90% of the patients [53]. According to the findings of a study that compared the outcomes of ASA or septal myectomy for hypertrophic cardiomyopathy in patients aged 65 years and younger to those aged ≤65 years old, patients aged 65 years and younger who underwent septal myectomy had higher in-hospital mortality than patients aged 65 years and older who underwent septal myectomy [54]. According to the findings of a comprehensive study, septal reduction treatments might be utilized as an alternative to medication therapy if the latter does not prove to be effective [55]. In their study, Guo *et al.,* recruited 226 individuals over 18 with hypertrophic cardiomyopathy and LVOT obstruction. In this group, 68 (31.1 percent) participants underwent percutaneous transluminal septal myocardial ablation (PTSMA), and 158 (69.9 percent) received modified Morrow Myectomy (MMSM). The patients selected for ablation were older adults who were more likely to need surgical intervention, given their advanced age. Moreover, LVOT pressure gradient may be relieved by both treatments and heart function could subsequently improve in individuals with HCM. However, as compared to PTSMA, MMSM may be able to reduce the angles more reliably [46]. In clinical trials, both intervention methods are effective in decreasing LVOT obstruction and alleviating patient symptoms. In contrast, it has been demonstrated that MMSM is superior to PTSMA in terms of lowering the gradient of LVOT obstruction and increasing the symptoms caused by HCM [52-58]. Another study with 51 consecutive patients with HOCM who underwent ASA found that older individuals experienced the same advantages as younger patients following the procedure; however, they experienced greater mortality events than younger patients [59]. Liebregts *et al.* reported data consistent with the aforementioned findings [32, 33, 40, 42, 46, 49, 50, 52-63].

### 3.3 Risk Includes the ASA’s Technological Capabilities

Veselka and colleagues recently investigated the approaches used to deliver ASA and observed that patients with HOCM who were given either a low dosage of alcohol (1.0–1.9 mL) or a high dose (2.0-3.8 mL) had comparable short and long-term results when given the same amount of alcohol. The low-dose alcohol group had a higher incidence of recurrent septal reduction surgeries and needed more procedures to be performed [64]. Sawaya *et al.* discovered that the transradial technique for alcohol septal ablation might be effective in the short- and long-term periods with fewer vascular problems than the transfemoral route [65]. One important deduction from the current analysis was the need for centers that perform ASA to have substantial prior experience in performing the procedure to reduce the likelihood of complications and maximize the therapeutic benefits [28, 51]. Mestres and colleagues confirmed that ASA is an alternative to surgical myectomy and has comparable effectiveness with respect to long-term survival [15, 16, 56]. Patients at high risk for surgery due to the co-morbidities or those who do not need cardiac surgery for any other concurrent pathology but otherwise fulfill the eligibility requirements for surgical intervention are urged to explore ASA as a viable option [16]. According to Yandrapalli *et al.*, patients undergoing ASA for HOCM had substantially lower mortality and hospitalization costs and length of stay (LOS) than those undergoing septal reduction (SR) for HOCM. ASA was associated with similar short-term outcomes, including mortality and 90-day readmission rates, but substantially lower hospitalization costs and LOS than SR. Taking together index admissions and readmissions, patients undergoing ASA had significantly lower LOS and costs [47].

## 4 RADIOFREQUENCY MYOCARDIAL SEPTAL ABLATION

### 4.1 Radiofrequency Ablation Safety and Efficacy

Radiofrequency myocardial septal ablation is an invasive therapeutic choice for individuals with drug-refractory symptoms of HOCM and left ventricular obstruction of less than 50 mmHg. It is a developing method that may be employed as a septum reduction therapy. Considering that it is less invasive than myectomy and alcohol ablation, this operation is possible for patients who are otherwise at high risk of complications from surgery [9, 10].

The application of radiofrequency energy, which induces resistive and conductive heating in the tissue, allows this treatment to generate lesions in the heart with well-defined boundaries. The target tissue is involuted as a result of the healing process. Current practice dictates that the surgery should be conducted under anesthesia, with cardiac electrical activity, blood pressure, and oxygen levels continuously monitored during the treatment. Additionally, there is a need for the installation of a temporary pacemaker. Finally, radiofrequency energy is delivered to the intraventricular septum *via* a percutaneous intramyocardial approach using a needle or catheter with a radiofrequency electrode. The procedure is guided by computed tomography angiography and an echocardiography, respectively [9, 10].

To determine its efficacy and safety, radiofrequency percutaneous cardiac septal ablation was studied in a group of 15 individuals with HOCM that had an average age of 40.7 years with 87% male distribution. During rest and exercise, the median outflow tract gradient of the heart was lowered by more than half, from 88 to 11 mmHg. The mitral regurgitation volume decreased from 4.3 mm to 0.5 mm, and pro-BNP was lowered from 924 mm to 137.5 mm [10].

Patients were subjected to cardiac resonance imaging after the radiofrequency intervention, which verified the ablation region with practical effects, including a decrease in fibrosis and septum. Most patients survived and exhibited improvement in the NYHA functional class, total exercise duration, and heart function after 6 months of follow-up. It should be noted that none of the patients had bundle branch block or total atrioventricular block during or after the surgery, as previously reported [10]. Patients with HCM and left ventricular apical aneurysms were studied by Igarashi *et al.* to determine the efficacy of radiofrequency catheter ablation for them. They found that the outcomes were favorable in 15 patients. Despite the successful suppression of ventricular tachycardia in all the patients with ablation, only two individuals had a recurrence of the condition, and one patient needed a second ablation [66].

After evaluating 11 patients with symptomatic HOCM requiring septal reduction who underwent radiofrequency ablation, Crossen and colleagues found that 10 had significant and persistent reductions in LVOT gradients at rest and when provoked after the procedure [9].

Valdigem *et al.* performed radiofrequency ablation on 12 patients with symptoms resistant to medical therapy. Similar to the previously described study, a drop in LVOT was found during the follow-up of patients after treatment, with a reduction in the mean of the maximum gradients from 96.8 34.7 mmHg to 62.7 25.4 mmHg in 3 months and from 96.8 34.7 mmHg to 36.1 23.8 mmHg in 1 year [52].

Further evidence from more recent investigations has revealed that the septal hypokinesia method is efficient in lowering LVOT gradients through the mechanism of septal hypotension [67-69]. Furthermore, Yang *et al.,* conducted a comprehensive study wherein they concluded that radiofrequency septal ablation successfully lowered LVOT gradients, which corroborated the finding that the decrease of the LVOT gradient by this method may be more significant [70].

Further, NYHA functional scores increased from a class mean of 3.00.0 to 1.80.8 following ablation, and symptoms improved following the procedure [68]. Further findings of a systematic review by Yang *et al.* demonstrated that ablation treatment improved NYHA functional class and alleviated symptoms associated with cardiac hypertrophy in all the trials [70].

A study by Liu *et al.* on 20 patients revealed consistent findings with the aforementioned studies and demonstrated an improvement in the NYHA functional class and a decrease in the LVOT gradient. This supports the hypothesis presented in the current study. Individuals with confined basal septal hypertrophy, shorter anterior mitral leaflet, and papillary muscles that were in the right position had much lower LVOT gradients than those who had other features [71].

Radiofrequency septal ablation is a safe procedure with the advantages of being less invasive and requiring a shorter hospital stay. Further, it can be performed in critical cardiac tissues with a high degree of hypertrophy and provides the opportunity to perform reapproach with new ablation when necessary, regardless of the underlying arterial anatomy. In addition to being readily accessible to older adults with various comorbidities and those with symptoms resistant to pharmacological therapy, it is also an effective treatment [9, 10].

It is important to emphasize that radiofrequency septal myocardial ablation should only be conducted in facilities with competent doctors who are well-versed in the cause of the left ventricular obstruction to prevent the necessity for a new strategy [9]. It has also been demonstrated by Liu *et al.* that using computed tomography angiography and echocardiography during the treatment boosted the overall safety of the procedure [10].

Considering that this technique does not require significant incisions or chest drains, wound-related complications are uncommon. Consequently, patients do not have to endure prolonged hospitalizations or other discomforts. It is essential to point out that likewise, recovery-related issues are also uncommon [9]. Crossen *et al.* stated that there were no long-term difficulties related to radiofrequency ablation and that there was also no significant morbidity linked with its use. Although some studies show that atrioventricular blocks and cardiac tamponade are potential consequences of this approach, other data indicate that these outcomes are unlikely to occur [64-72]. Accordingly, the study by Shelke *et al.* suggests utilizing multimodal pictures while the ablation procedure is being conducted to avoid these problems. This is consistent with the findings by Liu *et al.*, which suggest using imaging tests to increase safety [69-71].

In all eight investigations, researchers found that the patients’ mean resting LVOT gradient was considerably lowered. However, the average drop in this parameter ranged from 38.2 to 71.5 mmHg, depending on the study. The overall decrease in the LVOT gradient was 58.8 mmHg (*p*<0,01), and there was no statistically significant between-study heterogeneity. This was determined using a pooled analysis of all eight investigations. After radiofrequency ablation, the induced LVOT gradient decreased by 105.7 mmHg, according to the findings of another pooled study that included three separate investigations [70, 73, 74].

Cooper *et al.* investigated the use of a novel technology to guide radiofrequency ablation and found it effective. In this investigation, five patients received procedures using an imaging method called CARTOSound^®^ (Biosense Webster, Inc., USA) in conjunction with intracardiac echocardiography. This combination allowed for a more accurate mapping of the radiofrequency region while avoiding injury to the conduction tissue during the mapping process. Consequently, an improvement in the LVOT gradient was noted, with the gradient decreasing from 64.2 (50.6) to 12.3 (2.5) mmHg at rest and from 93.5 (30.9) to 23.3 (8.3) mmHg during exercise, respectively. Additionally, the NYHA status of the patients improved [75].

### 4.2 Complications from Radiofrequency Ablation

Only six clinical trials reported significant problems, while other clinical studies either did not record any issues or only documented a moderate consequence. Among 91 patients, two fatalities were reported caused by the surgery itself. One of these deaths was due to retroperitoneal hemorrhage, and the other was caused by a paradoxical rise in LVOT gradient produced by tissue edema after ablation. Further, eight patients with permanent pacemaker reliance had complete heart block, and three suffered VF requiring cardioversion [66-68]. This represented 8.8% of all the patients with persistent pacemaker dependency.

Recent clinical research on radiofrequency ablation has demonstrated that the electroanatomical mapping technique minimizes the incidence of pacemaker dependence [67-71, 73]. Following radiofrequency ablation, the conduction tissue, the left bundle, and the left anterior and posterior fascicles were directly traced. This allowed for the maintenance of atrioventricular conduction. Despite this, further study is required to demonstrate the advantages of radiofrequency septal ablation. The periprocedural mortality rate was similar to that of ASA and myectomy (around 2.2 percent). One of the 91 people with two instances of paradoxical elevation of the LVOT gradient passed away due to their experience. The administration of periprocedural dexamethasone may reduce the amount of tissue edema at the location of the ablation; however, further clinical study is needed to evaluate the benefit of this strategy, as elucidated in Table **1** [9, 70, 74-76].

## 5 GENE THERAPY

Multiple studies have elucidated the genetics of HCM [77-79], such as the influence of environmental variables on phenotypic features. Gene therapy is an appealing therapeutic option for HCM since it can potentially cure the condition. In this regard, the mutated *MYCB3* gene is gaining attention, and it has been associated with heart failure in pediatric patients and mortality. Promising results in murine models and human pluripotent stem cell-derived cardiomyocytes have been demonstrated by employing an adenovirus vector that transmits functional *MYCB3* to restore function [11, 69, 79-84].

Patients with double or compound heterozygosity (two variations in the same or separate sarcomere genes) had an earlier onset and worse disease progression [85]. Gene therapy methods have advanced during the previous decade. Genome editing, allele-specific silencing, spliceosome-mediated RNA trans-splicing, exon skipping, and gene substitution are a few examples [84]. Adeno-associated viruses (AAV) are used in cardiac gene therapy since they are low-pathogenic [86]. For HCM-associated MYBPC3 mutations, gene substitution using AAV9 seems promising. Mice and human pluripotent stem cell-derived cardiomyocytes also work [87-89]. Before human trials, AAV dosage and delivery must be tested in big animal models. A chosen group of hereditary HCM patients may benefit from this treatment [87-89].

Insights into the molecular genetic basis of HCM and advanced sequencing technology have allowed the more feasible, gene-based diagnosis of HCM. Genetic testing of HCM previously relied on polymerase chain reaction (PCR) amplification and sanger sequencing of amplicons of HCM genes [90]. Some clinical laboratories developed the gene chip platform using oligo hybridization-based sequencing technology [90, 91]. Recently, NGS technologies have been widely adopted; hence NGS-based HCM genetic testing has become more available in academic and commercial settings. Patients in whom the diagnosis of HCM is established, or suspected, should undergo clinical genetic testing that has been included as a reasonable approach to the diagnosis of HCM [92]. If an index case tests positive for a pathogenic or likely pathogenic variant, cascade screening in family members is recommended. The genetic diagnosis of HCM enables the accurate identification of preclinical variant carriers (genotype-positive/phenotype-negative), which warrant clinical evaluation and surveillance. At-risk individuals who have genotype-negative results no longer require routine electrocardiography and echocardiography. In addition, genetic testing allows for the appropriate reclassification of patient subsets with unrecognized phenocopy conditions such as Fabry disease, where enzyme replacement therapy can halt disease progression and complication [90].

The severity of symptoms, as measured by age at diagnosis, including the presence of a family history of HCM or SCD and maximal LV wall thickness, is greater in genotype-positive individuals than in genotype-negative patients, according to recent genotype-phenotype correlations [93]. The importance of genotypes in assessing patient prognosis and guiding clinical management in HCM was highlighted by findings from a large multicenter cohort showing that the presence of a sarcomere mutation is associated with earlier disease onset and serves as a strong predictor of adverse clinical outcomes, such as ventricular arrhythmia and HF [94]. There are several limitations to current genetic testing for HCM [95], even though it gives a definite molecular diagnosis and has the potential to save medical expenditures from recurrent clinical examinations. Well-validated gene panels are necessary to prevent genetic misdiagnosis since several genes often included in HCM gene panels may not have adequate evidence of disease connection. Clinical genetic testing may not be able to detect disease-causing mutations in 50% of patients with HCM (genotype-negative) owing to unknown genetic origin, possible somatic alterations, or acquired cardiomyopathy mimicking HCM. Identifying a genetic etiology for this clinically significant subset of HCM is crucial for realizing the full promise of genetic testing for HCM.

## CONCLUSION

Septal myectomy has been a popular choice since its related mortality and morbidity rates have decreased in recent years. Despite its positive results and the substantial study data amassed over four decades, the values and morbidity rates of surgical myectomy remain controversial. Transapical septal myectomy is an effective and safe treatment option for midventricular obstruction (MVO) in patients with hypertrophic cardiomyopathy. While further studies are needed to evaluate its efficacy and long-term outcomes, the available evidence suggests that TSM may be a suitable alternative to traditional septal myectomy for patients with MVO. Patients with symptomatic HOCM who are older and have comorbidities are candidates for alcohol septal or radiofrequency septal ablation. Alcohol septal ablation (ASA) is a minimally invasive procedure that involves injecting alcohol into a small artery that supplies blood to the thickened septum in patients with hypertrophic cardiomyopathy (HCM). ASA aims to reduce the thickness of the septum and improve blood flow, thereby relieving symptoms such as shortness of breath, chest pain, and fainting. ASA has positive effects such as improved symptoms, reduced left ventricular outflow tract obstruction, avoidance of surgery, and variable outcomes. However, ASA has several complications, such as arrhythmias, heart block, and coronary artery dissection. Limited long-term data are not well established, and there is a lack of randomized controlled trials comparing ASA to other treatment options for HCM. ASA may also be associated with an increased mortality risk compared to surgical myectomy. Percutaneous therapies are safe and effective and do not need extracorporeal circulation, which decreases postoperative pain and problems and allows for a second shot at the target. In situations with HOCM that do not respond to medication, radiofrequency septal ablation may be a viable invasive therapeutic option, as shown by the results. Radiofrequency ablation (RFA) is an effective treatment option for patients with hypertrophic cardiomyopathy (HCM) who have symptoms related to ventricular arrhythmias. Studies have shown that RFA can improve quality of life and reduce the risk of sudden cardiac death. It is minimally invasive and can be performed using a catheter inserted through a vein in the groin. However, it carries a risk of complications such as bleeding, perforation of the heart, and damage to surrounding structures. Limited efficacy and long-term data are not well established, and there is a lack of randomized controlled trials comparing RFA to other treatment options. It is important to consider the potential risks and limitations of the procedure when making treatment decisions. For further information on the effectiveness and safety of radiofrequency septal ablation in patients with HOCM, a large-scale randomized controlled study is required. More than three decades have passed since the first genetic etiologies were discovered; throughout this time, precision medicine-based therapies for genetic cardiomyopathies have developed to be rapidly implemented. Proximal medicines, such as gene therapies and main disease pathway modulators, have the potential to greatly influence the disease burden since they are most specific for a particular genetic etiology. However, modulators of secondary disease pathways may only affect a single effect of genetic variations that leads to detrimental cardiac remodeling, making them less selective.

## Figures and Tables

**Fig (1):**
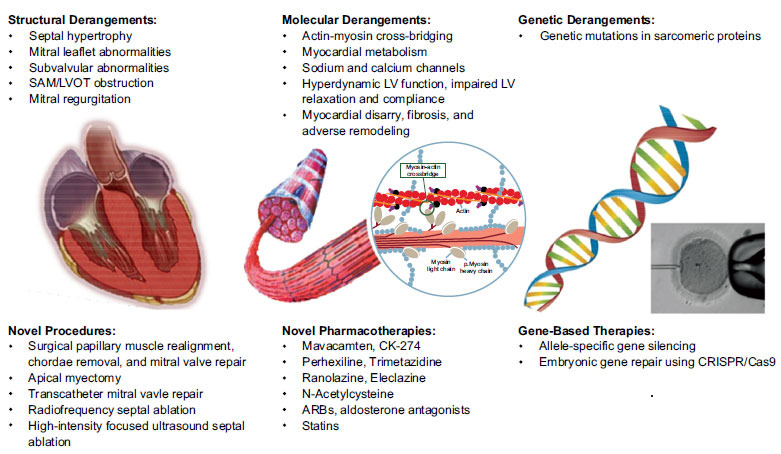
Novel therapeutic targets in hypertrophic cardiomyopathy. (Left) Novel procedural approaches target cardiac structural abnormalities in hypertrophic cardiomyopathy. (Middle) Novel pharmacotherapies target abnormal cellular processes in hypertrophic cardiomyopathy. (Right) Allele-specific gene silencing and genome editing using CRISPR/Cas9 target the genetic underpinnings of hypertrophic cardiomyopathy. **Abbreviations:** ARB, angiotensin II receptor blocker; LV, left ventricular; LVOT, left ventricular outflow tract; SAM, systolic anterior motion. Source: Tuohy CV, Kaul S, Song HK, Nazer B, Heitner SB. Hypertrophic cardiomyopathy: the future of treatment. Eur J Heart Fail. 2020;22(2):228-240. doi:10.1002/ejhf.1715 Order Date12-May-2022 / Order License ID1220726-1 / ISSN1388-9842

**Fig. (2):**
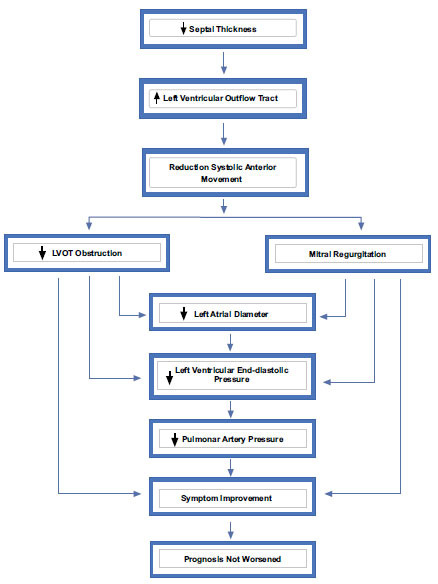
Evolution after percutaneous treatment with septal myocardial ablation. LVOT left ventricular outflow tract.

**Table 1:** Efficacy and complications of radiofrequency septal ablation.

**First Author/References**	**Year**	**Efficacy**	**Complications**
Crossen *et al.* [10]	2016	Persistent LVOT gradient decreases at rest and when provoked.	None.
Liu *et al.* [11]	2018	LVOT gradients were significantly and persistently reduced in 10 individuals.	All 9 individuals with ventricular ectopic beats recovered. One patient had pericardial tamponade.
Valdigem *et al.* [52]	2020	Patients’ post-therapy LVOT decreased.	None.
Zhou *et al.* [77-81]	2022	The therapy improved NYHA functional cardiac scores and symptoms.	Five patients had persistent right bundle branch block, two had resuscitated ventricular fibrillation, and two had septal branch aneurysms. Two in-hospital fatalities and 14 pericardial effusions occurred.
Yang *et al.* [70]	2020	Observed improvement of NYHA class.	Two procedure-related deaths, and eight patients had complete heart blocks.
Liu *et al.* [71]	2021	The observed improvement in the NYHA functional class as well as a decrease in the LVOT gradient.	One patient presented a transient complete atrioventricular block as a complication.
Qian *et al.* [82]	2021	The patients’ mean resting LVOT gradient was considerably lowered.	Two patients developed premature ventricular beats in the form of bigeminy during the procedure. Two other patients developed intraventricular block after treatment, but both recovered.
Cooper *et al.* [75]	2016	Improvement in the LVOT gradient was noted at rest and during exercise, respectively. Additionally, the NYHA class improvement.	One patient had a retroperitoneal hemorrhage, later evolving to death, and one patient had pulmonary edema as a complication.
Shelke *et al.* [69]	2014	Patients’ mean resting LVOT gradient was considerably lowered.	One patient developed pulmonary edema immediately after the procedure.
Lawrenz *et al.* [83]	2021	67% reduction in the LVOT gradient at rest after radiofrequency ablation and a 73% reduction after provocation.	A paradoxical increase in obstruction, and one of the patients died.
Lawrenz *et al.* [67]	2011	Reduction of 62% of the gradients at rest and 60% of the gradients caused by the LVOT.	None.
Shelke *et al.* [69]	2016	A sustained and significant reduction in LVOT gradient from baseline was observed in all but one patient at each follow-up. No post-procedure LVEF was worsening. Symptomatic status improved by at least one NYHA class in all patients, except in one.	One patient developed acute pulmonary edema immediately after the procedure.

**Abbreviations:** LVOT: left ventricular outflow tract; NYHA, the New York Heart Association. LVEF: Left Ventricular Ejection fraction.

## Data Availability

The article’s data will be shared on reasonable request to the corresponding author [A.M].

## LIST OF ABBREVIATIONS

HOCM: Hypertrophic Obstructive Cardiomyopathy
AF: Atrial Fibrillation
HCM: Hypertrophic Cardiomyopathy
LVOT: Left Ventricular Outflow Tract
ASA: Alcohol Septal Ablation
NYHA: New York Heart Association
SCD: Sudden Cardiac Death
ICDs: Implantable Cardioverter Defibrillators
PTSMA: Percutaneous Transluminal Septal Myocardial Ablation
VT: Ventricular Tachycardia
VF: Ventricular Fibrillation
LOS: Length of Stay
SR: Septal Reduction
MV: Mitral Valve
SAM: Systolic Anterior Motion
MR: Mitral Regurgitation
AAV: Adeno-associated Viruses
MVO: Midventricular Obstruction
